# *QuickStats*: Percentage* of Children and Adolescents Aged 6–17 Years Who Have Roads, Sidewalks, Paths, or Trails Where They Can Walk or Ride a Bicycle,^†^ by Urban-Rural Status^§^ and Family Income^¶^ — National Health Interview Survey, United States, 2020**

**DOI:** 10.15585/mmwr.mm7135a5

**Published:** 2022-09-02

**Figure:**
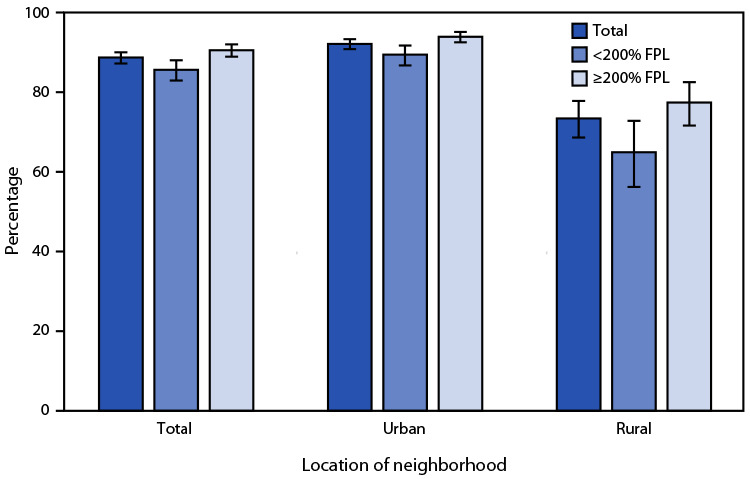
During 2020, 88.7% of children and adolescents aged 6–17 years had roads, sidewalks, paths, or trails in their neighborhood or near their home where they could walk or ride a bicycle. Availability of these spaces was less common among children and adolescents who lived in families with incomes <200% of FPL (85.6%) than among those in families with incomes ≥200% of FPL (90.5%) and was consistent among children and adolescents in both urban (89.4% versus 93.9%) and rural (64.9% versus 77.4%) areas. Regardless of income, availability of spaces to walk or ride a bicycle was lower among children and adolescents living in rural areas (73.4%) than among those in urban areas (92.1%).

**Source:** National Center for Health Statistics, National Health Interview Survey, 2020. https://www.cdc.gov/nchs/nhis/index.htm

